# Effect of Granite Residue Incorporation on the Behavior of Mortars

**DOI:** 10.3390/ma12091449

**Published:** 2019-05-05

**Authors:** Afonso Rangel Garcez de Azevedo, Markssuel Teixeira Marvila, Laimara da Silva Barroso, Euzébio Bernabé Zanelato, Jonas Alexandre, Gustavo de Castro Xavier, Sergio Neves Monteiro

**Affiliations:** 1 Department of Agricultural Engineering and Environment, Federal Fluminense University—UFF, Rua Passo da Pátria, 156, São Domingo, Niteroi, Rio de Janeiro 24210-240, Brazil; afonso.garcez91@gmail.com; 2 Laboratory of Civil Engineering, State University of North Fluminense—UENF, Avenida Alberto Lamego, Campos dos Goytacazes, 2000, Rio de Janeiro 28013-602, Brazil; laimarabarroso@hotmail.com (L.d.S.B.); ebzanelato@gmail.com (E.B.Z.); jonas.uenf@gmail.com (J.A.); gxavier@gmail.com (G.d.C.X.); 3 Department of Materials Science, IME—Military Institute of Engineering, Square General Tibúrcio, 80, Rio de Janeiro 22290-270, Brazil; snevesmonteiro@gmail.com

**Keywords:** mortar, granite residue, rheological

## Abstract

Civil construction is one of the most resource-consuming sectors in the world. For this reason, the last years have witnessed the study of reusing industrial residues in building materials. The ornamental stone processing industry has a considerable environmental liability related to residue generation during the cutting stages of granite blocks. The objective of this work is to analyze the viability of incorporating granite residues, up to 100%, to substitute sand in coating mortars for building construction. Mortars without residue, as control, and incorporated with 20, 40, 60, 80, and 100% of granite residue were subjected to consistency tests, incorporated air and water retention together with the rheological characterization using the squeeze-flow and the dropping-ball methods. The results show that mortars with 40% granite residues presented greater plastic deformation, helping their applicability by also presenting improved technological properties in the fresh state.

## 1. Introduction

In the current scenario, where there is a huge generation of residues on a global scale, it becomes essential that the construction industry be concerned with sustainable development, seeking alternatives to reuse solid wastes by reinserting them into the sector. In view of this, many studies are carried out with the aim of reducing the problems related to the destination of residues and minimizing the consumption of natural resources [1,2]. Recent research has evaluated the application of industrial solid residues in ceramic matrices, as a way of improving its technological properties [3]. Alternatives have been proposed for residues, such as cement matrix additions including concretes and mortars [4,5], as well as fillers in blocks of cement soil [6,7].

Ornamental stones’, such as granite, processing industry generates a large amount of residues during the process of transforming raw blocks (extracted in blast processes) into slabs. Up to 80% of loss occurs from original rocks, with other steps such as cutting and polishing [8,9,10] also contributing to more residues.

According to the Brazilian Association of Companies and Events (2015), Brazil was in 2017 the third largest exporter of granite in the world. The state of Espírito Santo is the producer of ~50% of Brazil’s ornamental stones and responsible for more than 70% of Brazil’s exports [6,11]. These high production numbers are accompanied by the generation of tons of residues, which need to be studied in order to create new alternatives for final disposal.

The behavior of cementitious materials, such as fresh mortars, depends on the variation in both the proportions of constituent materials and their quality, as well as the influence of the interfacial adhesion capacity between the constituents of the matrix [12,13]. In addition, in relation to viscosity and flow, when subjected to a certain shear stress, the mortar may have more extensive contact with the substrate, thus optimizing the adhesion mechanism [14,15]. The mortar needs a smaller coefficient of plastic viscosity, to reduce the work of densification and spreading. On the other hand, the drainage tension must be relatively high, since once applied to the wall, it should not drain, otherwise its applicability will be impaired [16,17]. Therefore, the study of mortars’ rheological behavior is of great relevance for the understanding of their behavior. An important methodology for the evaluation of mortar’s behavior, concerning its fluidity and consequent viability of use, is rheological evaluation—which estimates deformation according to the applied tension [18,19]. This term is of Greek origin (rheos = flow and logos = study). Rheology is the science that studies the flow and the tension of matter, evaluating the relations between applied shear tension and deformation in a certain period [20].

Some researchers have verified that a granite residue added to mortars in appropriate amounts is feasible and behaves as a filler, reducing the porosity of the matrix (material with silt and clay granulometry) and influencing other properties of the mortars in the fresh state, such as water retention and positively incorporated air [21,22,23]. Studies relating to the use of granite residue in cementitious materials (mortar and concrete) are described below [24,25,26,27]. López et al. [24] conducted studies to substitute micronized quartz powder for marble residue in high-performance concretes whose compressive strength is greater than 115 MPa. The authors performed the volume substitution of 35, 70, and 100% of the material. Based on the results obtained, Lopez et al. [24] concluded that the residue improves the workability properties and does not detract from the compressive strength parameters of the concrete studied. Chouhan et al. [25] conducted studies on the incorporation of a granite residue into concrete, where cement and fine aggregate are replaced by granite. The results demonstrated that a decrease in the workability parameters occurs when the substitution is performed for the cement, but the resistance parameters are improved. In the substitution of fine aggregate, the resistance parameters are generally maintained, as are the properties associated with the workability of the concrete. Gupta and Vyas [26] performed a study replacing fine aggregates by granite residues in cement mortars, while Mashaly et al. [27] performed a similar study but replacing cement in mortars by granite residues. The authors performed physical–mechanical tests, such as water absorption and compressive strength, as well as durability tests, concluding that the use of granite residues in mortar is technically feasible. However, both studies did not include in-depth study of the behavior of the mortars containing the residues, mainly with respect to rheology, which is the focus of our current study.

As granite residue generation increases, it becomes important to study the reuse of this material, in particular to analyze the feasibility of incorporating it into mortars. Thus, in this article different proportions of granite were used as substitutes for fine aggregates in mortar. Therefore, this work aims to analyze the viability of using a granite ornamental stone industry at different levels of substitution (20, 40, 60, 80, and 100%) for the fine aggregate of hydrated lime- and cement-based mortars. Residue free mortars were investigated as control samples.

## 2. Materials and Methods

The materials used in the present work were: (i) Portland cement CP II-E (with addition of blast furnace slag, traditionally used in Brazil due to the reduced marketing costs); and (ii) hydrated lime CH-III, which has a fineness modulus compatible with the use in coating mortars and contributes to the reduction of applied mortar retraction [28]. The nomenclatures are related to Brazilian standards ABNT NBR 16687:2018 [29] and ABNT NBR 7175:2003 [30]. Using the nomenclatures of the European standard the materials are classified as cement type CEM III (blast furnace cement) and hydrated lime type CL 80 (calcium aerial lime with 80% CaO and MgO content), in accordance to the norms EN 197-1:2011 [31] and EN 459-1:2010 [32], respectively. All commercial materials used (cement and lime) originated from the same supplied batch, thus reducing the possibility of variations, maintaining the homogeneity of the produced mortar.

The fine aggregate used was river sand from the city of Campos dos Goytacazes, Brazil, with standard granulometry for use in cementitious materials. The raw sand went obtained through a granulometry correction process by sieving. Drying was carried out in a greenhouse to remove all contained moisture in order to avoid interfering with the water / cement ratio of the cementitious composite.

The residue used for substitution of fine aggregate was obtained from the process of cutting and polishing blocks of granite from the city of Cachoeiro do Itapemirim, Brazil, an important national producer pole. The residue was carefully duly collected and transported in a humid form due to the use of water during the cutting process. Before use, the residue was sieved and homogenized in order to guarantee its uniformization.

Determining the proportion of mortar materials to be produced was based on studies aimed at optimizing the composition of the mixture [4,33,34]. This composition was calculated by volume, varying the granite residue and sand in each mortar (corresponding to 20% of the sand mass in the reference), resulting in a substitution ratio of 0, 20, 40, 60, 80, and 100%, according to Table 1. The composition chosen was 1:1:6, since it is used worldwide in coating mortars, according to the studies of Azevedo et al. [4], Marvila et al. [33], Azevedo et al. [34], and Zanelato et al. [35].

After determining the materials’ proportions, the mortar was carefully prepared in a controlled laboratory environment in order to standardize its properties, and to develop the main tests in a fresh and rheological state.

As for the physical characterization, both the sand and granite residues passed through a sieving process to determine the grain size curve, thus identifying if the aggregates were in the usable range, proposed by the Brazilian standard. In addition, the densities of sand and residue were determined, making the comparison possible [36].

The chemical characterization was performed to provide the composition of the material analyzed around its constituent elements and quantities. This was determined by X-ray Dispersive Energy Spectroscopy (XDS) using the Shimadzu EDX-70 equipment manufactured in São Paulo, Brazil. The morphological analysis of the granite residue by scanning electron microscopy (SEM) was also performed using the Shimadzu SSX 5550 equipment, manufactured in São Paulo, Brazil.

Environmental characterization was also performed, through leaching and solubilization tests, in order to evaluate the possible harmful effects that this exposed material (mortar) can cause to the environment.

The leaching test is a procedure used to separate substances contained in industrial waste by washing or percolation [37]. The leaching test was carried in the proposition 16:1 (solution:residue), using a deionized water solution and mechanical agitation. During the leaching test, the pH was maintained in the range of 5 ± 0.2. For pH control, 0.5 N acetic acid was used. Stirring was maintained in the sample for 24 h, and pH control was performed periodically throughout the test. The solubilization test was performed sequentially, where 250 g of greenhouse dried granite residue was used with 1000 mL of deionized and organic free water. The sample was shaken for 5 min, and allowed to stand for 7 days. The sample was then filtered and its pH recorded [38]. Both extracts obtained in the leaching and solubilization tests, were subsequently subjected to chemical analysis, allowing the correct identification.

The technological tests of fresh mortars were subdivided into consistency [39], air entrainment [40], and water retention [41], all fundamental importance for the understanding of the rheological behavior, together with the characterization analyses performed. All the highlighted tests were performed with three replicates per mortar composition.

The consistency to define the behavior of the mortar in fresh state is a necessary test to provide relations with its workability. This parameter is usually measured by means of the flow table test [39]. This test is a dynamic test where scales are applied by means of fixed height drops that cause the mortar to flow [4,17]. The consistency test comprises measuring the spreading of a quantity of molded mortar in the shape of a frustoconical (mold) on a table of consistency (flow table). It determines the amount of water necessary in order to confer characteristics of mixture applicability. The scattering should be limited to a range of 260 ± 5 mm to conform to the standards required for commercial use, as measured by a standard table in three diametric measurements [39].

Determining the incorporated air content within the evaluated traces was performed by filling a cylindrical metal mold of specified volume and known mass, suitably sized to be fully filled. Immediately after the mold containing mortar was weighed, the calculations were carried out to verify the mass density and later determine the incorporated air content of the mixture [40].

In order to determine the water retention capacity, the mortars were prepared and placed in a cylindrical mold of pre-established dimensions with a known mass. The surface was then regularized with the aid of a spatula, and the mold mass containing mortar was verified [41]. Two gauze screens were immediately placed on its surface, and twelve filter paper disks of known mass were placed on top of a metal plate, and a 2 kg loading was applied for 2 min. After this procedure, the mass of the filter paper disks containing a certain amount of retained water was determined. Once the calculations specified in the standard were made, water retention was determined [41].

Despite the complexity of the rheological behavior of mortars, they are traditionally characterized by simple tests, such as the flow table. Another suitable and appropriate methodology for the study of this property is the squeeze–flow test, which is a method for analyzing the behavior of mortar in relation to the deformation caused by an applied force [42].

**Figure 1:**
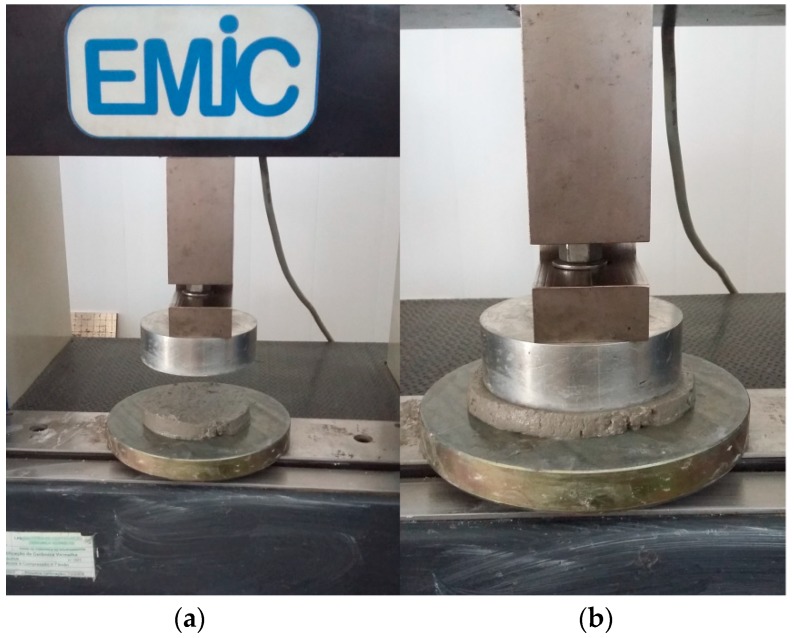
Exemplification of the rheology test by squeeze–flow. (**a**) Molded Sample and (**b**) compression in progress.

The test was performed in a universal EMIC test machine manufactured in São José dos Pinheiros, Brazil with adaptation to two parallel flat plates in which a compression load was applied to the mortar, causing a deformation at a constant displacement speed (Figure 1). A mortar sample with a diameter of 101 mm and a height of 10 mm was molded and a constant displacement speed of 0.1 mm/s was used for the slow type test [43,44].

**Figure 2:**
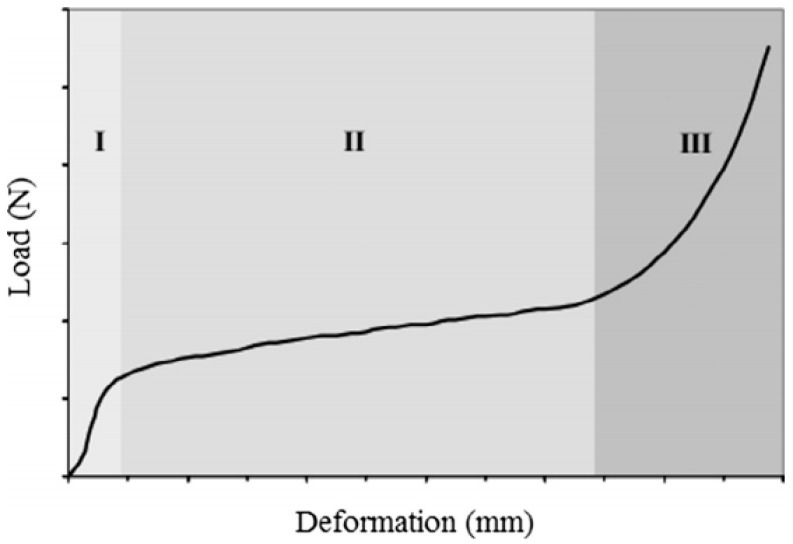
Typical profile of a force x displacement curve of a squeeze–flow test with displacement control. Source: Azevedo et al. [17].

The end of the squeeze–flow test occurs when the punch displacement of 9 mm or the maximum load of 1 kN is reached. The rheological results were expressed in graph of load (N) versus displacement (mm), following a standard profile of curvature in predefined regions, as indicated in Figure 2 [17].

Stage I is a small displacement showing the elastic deformation of the material, Stage II is an intermediate displacement showing plastic deformation or viscous flow, and Stage III is a large displacement and stiffening by deformation, influenced by the approximation of the aggregates and the friction formed by them [16,17].

**Figure 3:**
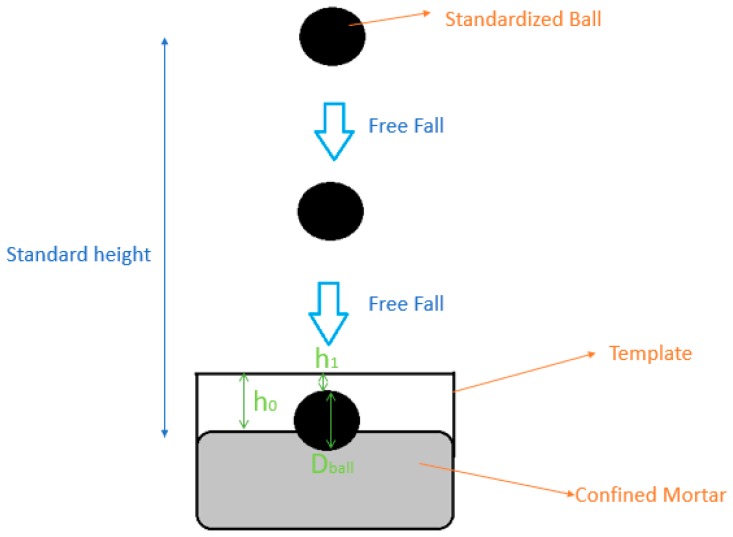
Exemplification of the rheology test by dropping-ball.

Finally, the dropping-ball test was performed using the British methodology [45]. This test is based on the free fall of a standard sphere (with fixed size, weight and drop height) on a mortar cast in a metal cylinder, as shown in Figure 3. The results are presented in the form of penetration index, a parameter directly related to the material flow stress, since the impact of the sphere applies an initial tension that tends to displace the mortar. The results of this test should be analyzed together with the incorporated air values of the mortars. If excess air is incorporated, the results of the dropping-ball can be impaired. The penetration index (mm) calculation was performed through Equation (1), which uses the parameters shown in Figure 3:

(1) $$PI=h_{1}+D_{ball}-h_{0}$$

## 3. Results and Discussion

### 3.1. Physical, Chemical, and Environmental Characterization

**Figure 4:**
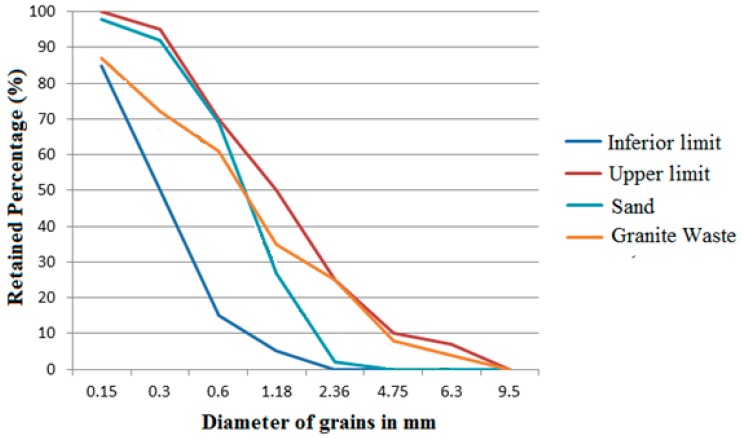
Result of the physical characterization of the fine aggregate (sand) and the granite residue.

Physical characterization is a fundamental guideline of the potentiality of residue incorporation, especially when it takes place in substitution (total or partial) of fine aggregates. This incorporation, besides seeking an improvement of the technological and rheological properties, contributes to the reduction of natural sand consumption, reducing the environmental impacts of the extraction process [10,11]. The result of the granulometric characterization, in comparative terms (residue and natural sand), can be observed in Figure 4. These results confirmed that both natural sand and granite residues are within the lower and upper range, normalized in Brazil, aiming to give adequate compaction to the cement matrix.

Granulometric arrangements in disagreement with the limits produce cementitious materials susceptible to greater degradation, as well as less internal cohesion between the constituents indicating mortars with high workability, which makes their commercial use inadequate [46]. Thus, granite residues meet the requirements, indicating initially a possible use in cementitious composites.

**Figure 5:**
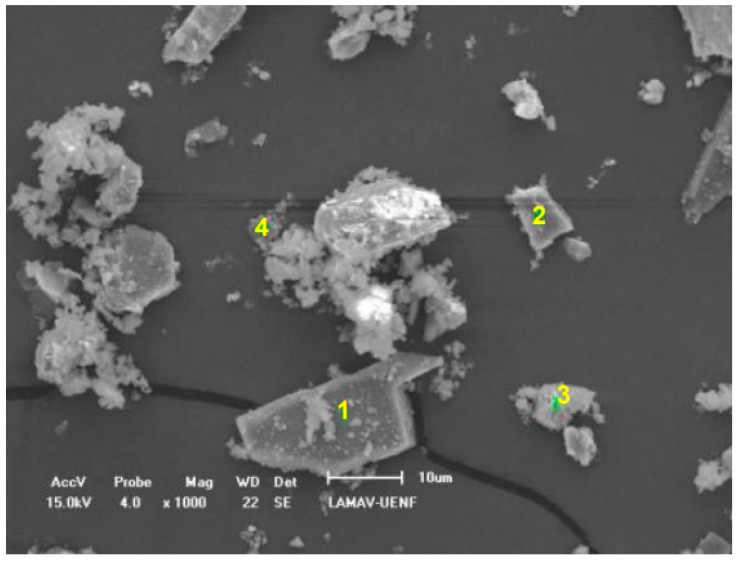
Morphological analysis of the granite residue by scanning electron microscopy (SEM).

The morphological analysis of the residue by scanning electron microscopy (SEM) is presented in Figure 5. It can be observed that the granite residue presents very irregular grains, since the process that gives origin to the material is the lamination that causes non-uniform changes in the material [6,9,10]. It is worth mentioning that, as widely reported in the literature [47,48], the natural sand extracted from the river presents its very regular morphology, with a circular and uniform shape. Thus, the morphology alteration promoted by the substitution of the residue caused changes in the technological properties of the mortar, as will be discussed in the sequence.

The specific gravity of sand and granite residues was 2.62 g/cm^3^ and 1.87 g/cm^3^, respectively, suggesting a tendency of larger voids internally in the granite residue, so much that its smaller mass in proportion to volume worked. The chemical evaluation of granite residues is presented in Table 2.

The results presented in Table 2 for silicon oxide (SiO_2_) in a proportion higher than 50% are a strong indicator of the presence of free silica and clay minerals such as kaolinite (Al_2_O_3_·2SiO_2_·2H_2_O), which have been verified by several studies [3,49]. The chemical composition presented is similar to granite rocks (such as quartz, feldspar and mica) [6].

The occurrence of Fe_2_O_3_ and CaO percentages comes from the beneficiation process, through iron plots used as raw abrasive cutting material extracted from nature [6,9,10]. The small loss to fire (LF) is due to the deterioration process of the mica present in the material [49]. Table 3 shows results of the granite residue leaching test.

Considering the results of Table 3, it can be observed that the concentrations are within the Brazilian normative standards and are compatible with the main international environmental conditions [50], and can be considered as a non-hazardous waste, which is a positive indication for its use [37]. The result of the solubilization extract analysis is given in Table 4.

According to Table 4, practically all chemical elements were within the limit of the Brazilian technical norm, except in the concentration of Pb that was slightly above the normative limit, indicative of the cutting process from which it is submitted [50]. This allows us to classify the residue as not inert, so the final residue is classified as non-hazardous and not inert.

### 3.2. Technological Characterization

The mortars were immediately submitted for homogenization for the main technological tests in the fresh state—determining the standard consistency, to estimate the amount of water needed in each trait (Table 5).

It can be concluded from Table 5 that the amount of water needed to ensure a consistency within the applicability standards of the mortar, causing spreading around 260 ± 5 mm, increased as the percentage of granite residue was replaced by the aggregate.

**Figure 6:**
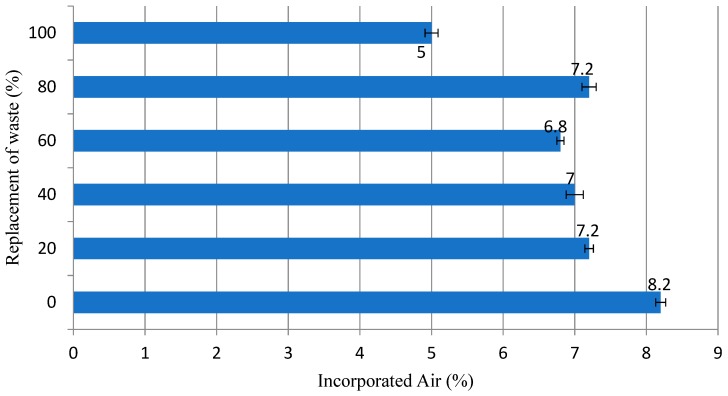
Result of the incorporated air content (%) in the evaluated mortars.

This increase was due to the granite residue, which unlike sand, has a greater water absorption potential due to its microstructure and its main chemical components, as analyzed in its characterization [21,22,23]. The proximity of the granulometric arrangement results in low quantities; otherwise the mortar would have an excess of fluidity, making it difficult to apply it in civil construction. Another technological analysis is the results of the incorporated air content, which plays a fundamental role in the other rheological properties, such as viscosity and mortar flow, the result of which can be observed in Figure 6 below [16,17].

Figure 6 shows that the substitution of natural aggregates for granite residues generated a reduction in the percentage and air incorporated in the mixture, a fact justified by the greater internal compactness of the matrix, due to residue processing promoting an arrangement with more uniform grains and played a role of filling better than natural sand, this may directly impact the mechanical strength of these mortars [46,49].

**Figure 7:**
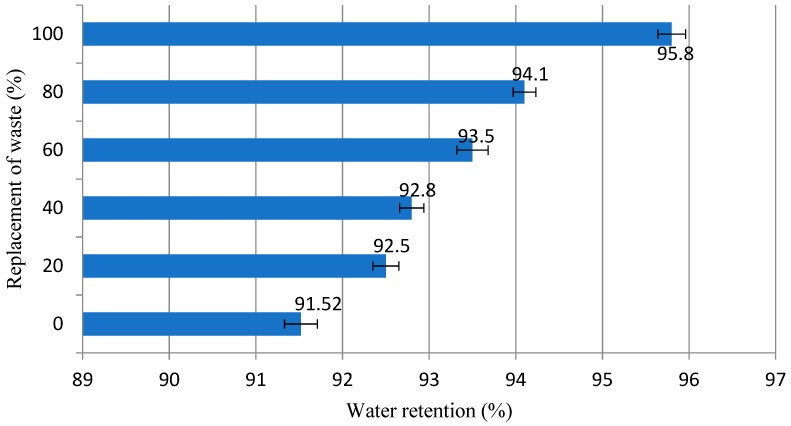
Result of water retention (%) of evaluated mortars.

However, excess compactness and lower values of incorporated air pose a great problem for the applicability of the mortar in buildings, so the trace of 100% presents little usable values, according to literature data [51,52]. During the execution of the technological tests, it was verified that the substitution of 100% presented recurring problems in the homogenization process, proving the results obtained by the incorporated air content. The water retention results are shown in Figure 7.

The results demonstrated in Figure 7 indicate that the addition of granite residue substitution caused an increase in the water retention, which is internally entrapped in the microstructure of the residue, provoking this increase [53]. High water retention values (above 90% according to the international bibliography [51,53]) improve the resistance parameters of the mortar, since water entrapment maintains favorable conditions for cement hydration [53,54,55]. On the other hand, very high retention values make it difficult to fix the mortar to the substrates, especially the ceramic ones, which leads to a reduction of the flow stress in the fresh state of the mortar [56]. Water retained in the mortar, which in fact should be considered as a paste containing cement particles, promotes micro anchoring of the mortar in the substrate [56,57], aiding properties such as adhesion and making the mortar more workable, since this material is fixed to the substrate. It is worth mentioning that mortars with a water retention content greater than 95% should be considered satisfactory. This fact makes it impossible to use mortars with 100% granite residue.

### 3.3. Rheological Characterization

**Figure 8:**
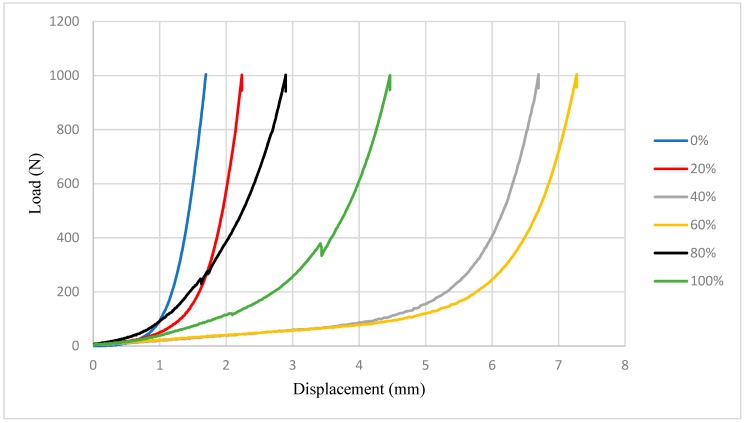
Result of the load x displacement curve at different mortar replacement levels.

The rheological characterization was performed using the squeeze–flow methodology, which measures the scattering after the compressibility of the mortar on a metal plate, the result can be observed in Figure 8.

Figure 8 shows that the trait without granite addition, denominated 0% presented elastic deformation (Stage I), in which it characterizes the behavior of a solid material and Stage III, in which an increase of the force is required for the deformations are constant, well defined. However, it obtained difficulty in stage II that presents the plastic deformation [16,17].

The mortar with 20% content presented a similar behavior to that of mortar of 0%, with greater difficulty of flow, leaving the initial stage, direct to the stiffening by deformation. This is because it presents high friction between the aggregates [17,18,19,20]. The curve of the 80% content also presented difficulty of flow, soon reaching the hardening by deformation. Based on these configurations, it is possible to notice that these compositions (20% and 80%), although similar to reference mortar, present handling difficulties because they present a behavior of a very viscous fluid with a mechanical behavior similar to that of a solid.

**Figure 9:**
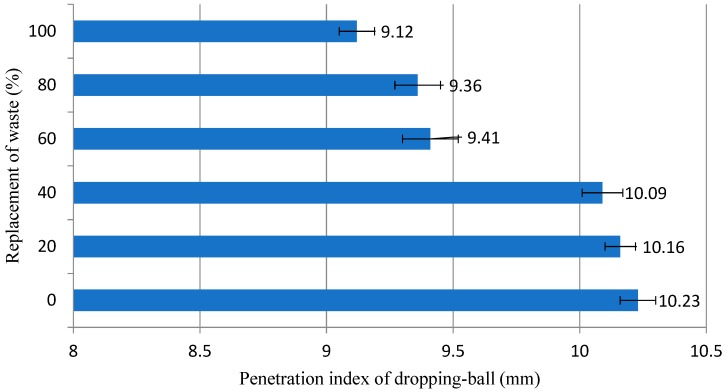
Penetration index results obtained by dropping-ball for mortars evaluated.

The contents of 40% and 60% presented a very similar behavior, with a great part of the plastic deformation and then reaching the stiffness due to deformation. The curve of the 100% content was the only one to pass clearly through the three stages, elastic deformation, plastic deformation, and stiffness due to deformation [17]. However, it should be noted that the composition with 100% of residues presented noises in the squeeze–flow curve, as observed in Figure 8 around 400 N, indicating that this mortar presented high internal friction [16], which compromises its workability parameters. The occurrence of internal friction in the composition with 100% granite residue can be explained by the morphology of the particles of this material, which as seen in Figure 5, is highly irregular, contributing to a noise in the squeeze–flow curve of this material. Based on these aspects obtained by the analysis of the squeeze–flow, it is possible to understand that the curves of 40% and 60% present the best rheological behavior. Figure 9 shows the penetration index results obtained by dropping-ball.

It is observed that, gradually, the incorporation of granite residues reduces the penetration rate of mortars. This fact confirms that the incorporation of the studied residue increases the flow stress of the mortars, a fact that damages the workability of the material, since the higher the flow stress, the more effort must be employed to cause the mortars to start moving. The increase of the flow stress is closely related to the morphological form of the sand grains and the grain of the residue (Figure 5). While the sand presents uniform rounded and regular grains, due to the weathering process that originates these grains [47,48], the granite residues are not uninform, irregular particles, without a standard format. Thus, mortars made predominantly with sand (composition 0%, 20%, and 40%) have high penetration rates, because the grain shape favors the initial movement of the mortars, while the mortars containing the largest proportions of granite (100%, 80%, and 60%) present difficulties to initiate the movement, evidenced by the low penetration indices that prove higher values of yield stress, since more effort is needed to break the inertia due to the stiffness caused by the grains of irregular shape. This fact discourages the use of mortars with residual contents above 60%.

## 4. Conclusions

Residues from granite processing have potential for application in cement matrices, according to its physical and chemical characterization, indicating that the reactive process with the cement paste is suitable for civil construction applications;

The waste studied was classified as non-hazardous and non-inert, this is a key indicator of applicability in other production chains, besides favoring a proper disposal and in an environmentally correct way;

As for the technological properties, the percentage of 100% does not present potential use in the analyzes made, such as consistency, water retention and incorporated air;

As for the rheological characterization, we found that in all the contents, tensile strength is present, but in the 40% and 60% substitutions the plastic deformation is predominant, favoring the productivity in the application of mortar, while the mortars with content of 0% and 20% presented greater difficulty of flow, thus making the procedures of material application and finishing difficult. At 80% content, a reduction in the loads was necessary for material deformation due to its rupture occurred and the sample with 100% presented deformation by the stiffening, but with presence of internal friction of its grains;

In the dropping-ball test the results lead to the conclusion that mortars with lower residue contents (0%, 20%, and 40%) present lower flow stresses since they present higher penetration rates. This fact is attributed to the characteristic morphology of the granite residue, which, because it is irregularity, increases the initial effort required for the mortar to start moving.

The 40% combination best combines the rheological and technological properties for the use of the granite residue in mortars, being corroborated by the physical, chemical, and environmental characterization of this work.

Finally, it is recommended that future studies focus on the interference of granite residue in other mortar properties, such as mechanical strength, water absorption, and porosity, to complement the understanding of mortar behavior with this material.

## Figures and Tables

**Table 1:** Compositions of mortars studied.

Replacement Levels (%)	Cement (CPII-E) (g)	Hydrated Lime (CH-III) (g)	Granite Residue (g)	Sand (g)
0	200.00	94.09	0.00	1361.00
20	200.00	94.09	272.20	1088.80
40	200.00	94.09	544.40	816.60
60	200.00	94.09	816.60	544.40
80	200.00	94.09	1,088.80	272.20
100	200.00	94.09	1,361.00	0.00

**Table 2:** Chemical composition of the granite residue (%).

SiO_2_	Al_2_O_3_	Fe_2_O_3_	K_2_O	TiO_2_	SO_3_	CaO	Na_2_O	BaO	Others	LF
63.23	15.34	3.53	5.34	1.13	1.52	3.34	3.04	0.34	3.19	1.34

LF = Loss to fire.

**Table 3:** Leaching test conditions and results for the granite residue.

Chemical Element	Leaching Allowed (mg/L)	Leaching Obtained for Granite Residue (mg/L)
Ag	5.000	0.0004
Cd	0.500	0.15
Cr	5.000	0.45
Pb	5.000	2.34
Ba	100.00	-

**Table 4:** Result of the solubilization test of the granite residue.

Chemical Element	Granite Residue (mg/L)	Maximum Solubility Allowed by the Brazilian Standard (mg/L)
Ag	<0.004	0.050
Cd	<0.001	0.005
Cr	<0.020	0.050
Pb	<0.065	0.050
Ba	<0.010	1.000
Al	0.130	0.200
Cu	<0.010	1.000
Fe	0.025	0.300
Mn	<0.010	0.100
Zn	<0.003	5.000
Na	33.900	200.000
Chlorides	9.570	250.000
Toughness	18.500	500.000
Sulfates	10.450	400.000

**Table 5:** Result of the amount of standard water in each mortar, through the consistency test.

Replacement (%)	Consistency Index (mm)	Amount of Water Added (g)
0	262.00	345.00
20	261.00	348.00
40	259.00	352.00
60	263.00	355.00
80	260.00	360.00
100	258.00	361.00
